# *Phyllachora* species infecting maize and other grass species in the Americas represents a complex of closely related species

**DOI:** 10.1002/ece3.8832

**Published:** 2022-04-25

**Authors:** Kirk Broders, Gloria Iriarte‐Broders, Gary C. Bergstrom, Emmanuel Byamukama, Martin Chilvers, Christian Cruz, Felipe Dalla‐Lana, Zachary Duray, Dean Malvick, Daren Mueller, Pierce Paul, Diane Plewa, Richard Raid, Alison E. Robertson, Catalina Salgado‐Salazar, Damon Smith, Darcy Telenko, Katherine VanEtten, Nathan M. Kleczewski

**Affiliations:** ^1^ Agricultural Research Service National Center for Agricultural Utilization Research Mycotoxin Prevention and Applied Microbiology Research Unit. 1815 N. University USDA Peoria Illinois USA; ^2^ Independent Data Analyst Dunlap Illinois USA; ^3^ 5922 Plant Pathology and Plant‐Microbe Biology Section School of Integrative Plant Science Cornell University Ithaca New York USA; ^4^ 2019 Department of Agronomy, Horticulture, and Plant Science South Dakota State University Brookings South Dakota USA; ^5^ 3078 Department of Plant, Soil, and Microbial Sciences Michigan State University East Lansing Michigan USA; ^6^ 311308 Department of Botany and Plant Pathology Purdue University West Lafayette Indiana USA; ^7^ Department of Plant Pathology and Environmental Microbiology Southeast Agricultural Research & Extension Center Pennsylvania State University Manheim Pennsylvania USA; ^8^ Department of Crop Sciences University of Illinois Urbana Illinois USA; ^9^ Department of Plant Pathology University of Minnesota St Paul Minnesota USA; ^10^ 1177 Department of Plant Pathology and Microbiology Iowa State University Ames Iowa USA; ^11^ 2647 Department of Plant Pathology Wooster The Ohio State University Ohio USA; ^12^ 3463 Department of Plant Pathology University of Florida Gainesville Florida USA; ^13^ Agricultural Research Service, Mycology and Nematology Genetic Diversity, and Biology Laboratory USDA Beltsville Maryland USA; ^14^ 5228 Department of Plant Pathology University of Wisconsin‐Madison Madison Wisconsin USA

**Keywords:** biotrophs, pathogen diversity, phyllachorales, phylogeny, sympatric speciation, tar spot

## Abstract

The genus *Phyllachora* contains numerous obligate fungal parasites that produce raised, melanized structures called stromata on their plant hosts referred to as tar spot. Members of this genus are known to infect many grass species but generally do not cause significant damage or defoliation, with the exception of *P*. *maydis* which has emerged as an important pathogen of maize throughout the Americas, but the origin of this pathogen remains unknown. To date, species designations for *Phyllachora* have been based on host associations and morphology, and most species are assumed to be host specific. We assessed the sequence diversity of 186 single stroma isolates collected from 16 hosts representing 15 countries. Samples included both herbarium and contemporary strains that covered a temporal range from 1905 to 2019. These 186 isolates were grouped into five distinct species with strong bootstrap support. We found three closely related, but genetically distinct groups of *Phyllachora* are capable of infecting maize in the United States, we refer to these as the *P*. *maydis* species complex. Based on herbarium specimens, we hypothesize that these three groups in the *P*. *maydis* species complex originated from Central America, Mexico, and the Caribbean. Although two of these groups were only found on maize, the third and largest group contained contemporary strains found on maize and other grass hosts, as well as herbarium specimens from maize and other grasses that include 10 species of *Phyllachora*. The herbarium specimens were previously identified based on morphology and host association. This work represents the first attempt at molecular characterization of *Phyllachora* species infecting grass hosts and indicates some *Phyllachora* species can infect a broad range of host species and there may be significant synonymy in the *Phyllachora* genus.

## Graphical abstract

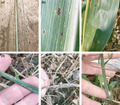

## 1 INTRODUCTION

**FIGURE 1:**
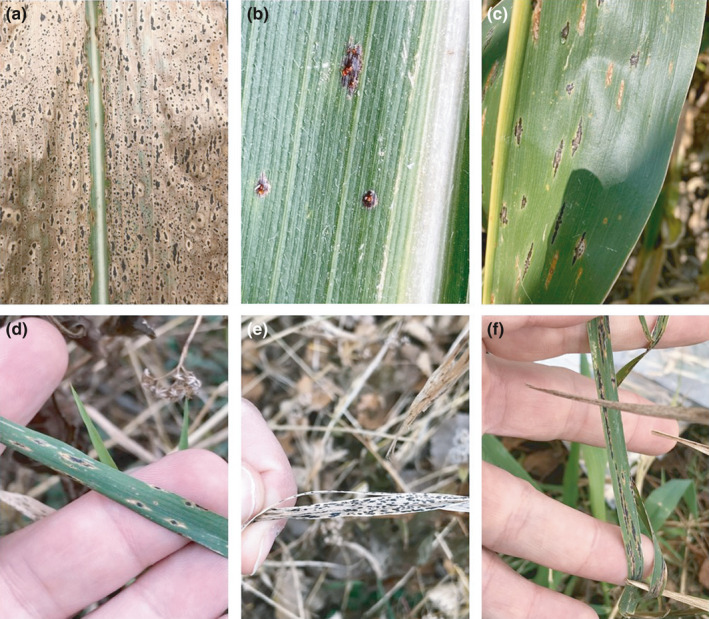
Signs and symptoms of *Phyllachora* spp. on grasses. *Phyllachora maydis* on maize at severe levels (a); with ascospores being extruded from stroma (b) and showing characteristic tapering ends of mature stromata (c). *Phyllachora* spp. on *Elymus* in Michigan (d), Fall Ryegrass in Illinois, and an unidentified grass in Indiana (f). Photo credit N. Kleczewski

Phyllachorales is a monophyletic order of biotrophic fungi comprised of approximately 1,226 recognized species (Maharachchikumbura et al., 2016; Mardones et al., 2017), but global estimates of species within this order approach 160,000 (Cannon, 1997). The Phyllachorales largely contain plant parasitic fungi and are commonly associated with monocotyledonous plants across a range of habitats. These fungi are often referred to as “tar spot” fungi due to the production of stromata on plant hosts that resemble black flecks of tar (Figure 1) (Mardones et al., 2017).

Tar spot of maize (Figure 1a–c), caused by the fungus *Phyllachora maydis*, emerged in the United States in 2015, with the disease expanding each year since the initial report and continuing to have a significant economic impact on maize across many production regions in the United States (Kleczewski et al., 2020; Valle‐Torres et al., 2020). Since first identified in North America in 2015, *P*. *maydis* has spread rapidly throughout the United States and Canada (Kleczewski & Bowman, 2020; Kleczewski et al., 2020), and resulted in yield losses exceeding $US 658 million in 2018 (Mueller et al., 2020). Although tar spot symptoms caused by members of the genus *Phyllachora* have been commonly observed on a number of grasses (Figure 1d–f) and shrub species throughout North, Central, and South America, historically the fungus has rarely been known to cause significant plant damage. However, tar spot has been occasionally reported to cause severe damage to maize in Mexico, Central America, and several Caribbean Islands (Valle‐Torres et al., 2020).

The origin of *P*. *maydis* within the United States is not currently known, although the presence of two distinct epicenters of maize tar spot in the Midwest and Southeast indicates at least two separate emergence events. While tar spot is a new disease on maize in the United States and Canada, it has been present in Mexico, several Caribbean islands including Puerto Rico, Cuba, and the Dominican Republic as well as Central American Countries, such as Guatemala, Honduras, Nicaragua, and Costa Rica for the last century but only caused limited damage. In addition, tar spot signs and symptoms caused by *Phyllachora* species are common on several native and weedy grass species in North America (Figure 1d–f) (Orton, 1944). The monographic work by Orton (1944) was completed solely by morphological identification and host affinity. Given our understanding of phenotypic plasticity of many fungi and the ability of biotrophic pathogens to infect multiple hosts (Morris & Moury, 2019), it is possible that cryptic species or species complexes may be present.

Species definitions within the Phyllachorales have historically been based largely on morphological characteristics and assumption of high host specificity due to their presumed biotrophic nature. However, there are examples in the genus where this assumption of host specificity does not hold true (Cannon, 1991, 1997). Furthermore, species designations based on host specificity are highly dependent on accurate identification of the host species, which may be difficult or impossible in some instances. For example, *P*. *graminis* (Pers.) Fuckel is considered a “dustbin” species where many specimens of isolates infecting grasses are deposited with the host not often identified to species (Parbery, 1967). Furthermore, factors such as nutrients available to the fungus, temperature, light quality, light cycles, substrate type, host, and epigenetic factors may also result in alterations in fungal morphology that may result in inaccurate species designations (Francisco et al., 2019; Money, 2013; Slepecky & Starmer, 2009; Stockinger et al., 2009). Thus, our current understanding of the genetic diversity, host range, and species delimitation within the genus *Phyllachora* is relatively limited and requires reevaluation.

The recent emergence of *P*. *maydis* in the United States and Canada may also be associated with the ability of the fungus to better persist and spread than previously thought. Once established, the fungus can survive at least one winter at subzero temperatures on corn residue as ascospores within stromata, which are believed to be the main inoculum source the following season (Groves et al., 2020; Kleczewski et al., 2019). Under periods of moderate temperatures and wet weather, it is believed that ascospores are dispersed by wind and rain splash where they land on the foliage, stalks, and husks of corn. After spore germination and infection of the host, the fungus remains dormant for at least 2 weeks after which stromata, and associated spermatia and ascospores, are produced. Data from Central America indicated a relatively steep dispersal curve of *P*. *maydis* ascospores from a source (Hock et al., 1995). However, the rapid spread of this fungus throughout the Midwest, coupled with observations of “top down” infestations in fields with no history of disease and observations of infestations of isolated plots located 1,200 m from potential inoculum sources, indicate that the pathogen can travel much further across local/regional topographies than estimated previously (Kleczewski et al., 2020).

Based on this information, the emergence of *P*. *maydis* on corn in the United States and Canada could have been the result of many factors including the introduction of the fungus on infected plant material, natural northern dispersal through wind, establishment in the United States favored by climate change, changes in hybrid genetics, a host jump from a grass species, or a combination of any of these four. This study represents the first attempt to extract and sequence DNA from *Phyllachora* stroma on fresh and herbarium‐infected grass specimens. The goal of this study is to understand the genetic diversity of *Phyllachora* species causing tar spot disease in contemporary corn production regions in the United States, and compare this to historical specimens of *Phyllachora* species from herbarium samples of maize and other grasses from Mexico, Central, and South America, the Caribbean, and Europe, as well as contemporary and herbarium species of *Phyllachora* species associated with grass hosts in the United States. This represents the first attempt at genetic characterization of *Phyllachora maydis*, *Phyllachora graminis*, and other grass infecting *Phyllachora* species. The objectives of this study are to: (1) determine if a single species of *Phyllachora* is responsible for tar spot disease of maize throughout its range in the Americas over the last century or if distinct genetic groups are responsible for these symptoms; and (2) determine if *Phyllachora* species infecting native and weedy grasses in close proximity to maize production fields are the same species as those infecting maize. Understanding the phylogenetic diversity and the potential host and geographic range of *Phyllachora* species associated with maize and other grasses in the Americas will also help to infer the potential evolutionary origins and speciation patterns in this genus.

## 2 MATERIAL AND METHODS

### 2.1 Sample collection

Samples of maize and wild grasses with characteristic stromata of *Phyllachora* spp. (Figure 1a–f) were collected from across North America and Mexico in 2018 and 2019 (Table 1). Field specimens of infested maize and other grasses were collected by numerous individuals from the agricultural community as described in Kleczewski et al. (2020). Samples were pressed, dried at room temperature, and stored at 20°C in manila envelopes until processed. Herbarium specimens were obtained from the U.S. National Fungus Collection (BPI, Beltsville, Maryland) and the University of Illinois Herbarium (Urbana, Illinois), which included specimens on maize and other grasses from additional hosts, countries, and years (Table 1). A total of 186 samples from 16 hosts and 15 countries, collected from 1905 to 2019, were included in the analyses.

**TABLE 1:** The sample ID, genetic cluster, geographic and host origin, year collected, and source of the 186 *Phyllachora* specimens used in this study

Sample ID	Genetic cluster^a^	Species^b^	State	Country	Host	Year collected	Source	GenBank accessions	
								ITS	LSU
BPI893226_2	1	*Phyllachora maydis*	Indiana	USA	*Zea mays*	2015	Field collection	OL342800	
BPI893227_2	1	*P. maydis*	Indiana	USA	*Zea mays*	2015	Field collection	OL342801	
BPI893229_2	1	*P. maydis*	Indiana	USA	*Zea mays*	2015	Field collection	OL342802	
BPI893230_2	1	*P. maydis*	Indiana	USA	*Zea mays*	2015	Field collection	OL342803	
BPI893231_2	1	*P. maydis*	Indiana	USA	*Zea mays*	2015	Field collection	OL342804	
BPI893232_2	1	*P. maydis*	Indiana	USA	*Zea mays*	2015	Field collection	OL342805	
C18001‐2	1	*P. maydis*	Indiana	USA	*Zea mays*	2018	Field collection	OL342781	
C18001‐3	1	*P. maydis*	Indiana	USA	*Zea mays*	2018	Field collection	OL342782	
C18003‐1	1	*P. maydis*	Indiana	USA	*Zea mays*	2018	Field collection	OL342783	
C18003‐2	1	*P. maydis*	Indiana	USA	*Zea mays*	2018	Field collection		OL314402
C18003‐3	1	*P. maydis*	Indiana	USA	*Zea mays*	2018	Field collection	OL342784	
C18009‐1	1	*P. maydis*	Indiana	USA	*Zea mays*	2018	Field collection	OL342785	
C18009‐2	1	*P. maydis*	Indiana	USA	*Zea mays*	2018	Field collection	OL342786	
C18011‐3	1	*P. maydis*	Indiana	USA	*Zea mays*	2018	Field collection	OL342787	OL314403
C18024‐1	1	*P. maydis*	Indiana	USA	*Zea mays*	2018	Field collection	OL342788	
C18024‐2	1	*P. maydis*	Indiana	USA	*Zea mays*	2018	Field collection	OL342789	
C18024‐3	1	*P. maydis*	Indiana	USA	*Zea mays*	2018	Field collection	OL342790	
C18161‐1	1	*P. maydis*	Ohio	USA	*Zea mays*	2018	Field collection	OL342791	OL314404
C18161‐2	1	*P. maydis*	Ohio	USA	*Zea mays*	2018	Field collection	OL342792	OL314405
C18161‐3	1	*P. maydis*	Ohio	USA	*Zea mays*	2018	Field collection		OL314406
C18162‐1	1	*P. maydis*	Ohio	USA	*Zea mays*	2018	Field collection	OL342793	OL314407
C18162‐3	1	*P. maydis*	Ohio	USA	*Zea mays*	2018	Field collection	OL342794	OL314408
C18164‐1	1	*P. maydis*	Ohio	USA	*Zea mays*	2018	Field collection	OL342795	
C18164‐2	1	*P. maydis*	Ohio	USA	*Zea mays*	2018	Field collection	OL342796	OL314409
C18164‐3	1	*P. maydis*	Indiana	USA	*Zea mays*	2018	Field collection	OL342797	
C19043‐1	1	*P. maydis*	Indiana	USA	*Zea mays*	2019	Field collection	OL342798	
C19043‐3	1	*P. maydis*	Indiana	USA	*Zea mays*	2019	Field collection	OL342799	
BPI638548_1	2	*P. maydis*	Cundinamarca	Colombia	*Zea mays*	1940	USDA Herbarium	OL342824	
BPI638554_1	2	*P. maydis*	Añasco	Puerto Rico	*Zea mays*	1917	USDA Herbarium	OL342825	
BPI638578_1	2	*P. maydis*	Vega Baja	Puerto Rico	*Zea mays*	1916	USDA Herbarium	OL342826	
BPI910562_1	2	*P. maydis*	Michigan	USA	*Zea mays*	2017	USDA Herbarium	OL342827	
C18026‐3	2	*P. maydis*	Puebla	Mexico	*Zea mays*	2018	Field collection	OL342806	
C18030‐1	2	*P. maydis*	Guerrero	Mexico	*Zea mays*	2018	Field collection	OL342807	OL314410
C18030‐2	2	*P. maydis*	Guerrero	Mexico	*Zea mays*	2018	Field collection	OL342808	OL314411
C18030‐3	2	*P. maydis*	Guerrero	Mexico	*Zea mays*	2018	Field collection	OL342809	OL314412
C18031‐1	2	*P. maydis*	Veracruz	Mexico	*Zea mays*	2018	Field collection	OL342810	OL314413
C18031‐2	2	*P. maydis*	Veracruz	Mexico	*Zea mays*	2018	Field collection	OL342811	OL314414
C18031‐3	2	*P. maydis*	Veracruz	Mexico	*Zea mays*	2018	Field collection	OL342812	OL314415
C18033‐2	2	*P. maydis*	Oaxaca	Mexico	*Zea mays*	2018	Field collection	OL342813	OL314416
C18033‐3	2	*P. maydis*	Oaxaca	Mexico	*Zea mays*	2018	Field collection	OL342814	OL314417
C18038‐3	2	*P. maydis*	Guerrero	Mexico	*Zea mays*	2018	Field collection	OL342815	
C18040‐1	2	*P. maydis*	Florida	USA	*Zea mays*	2018	Field collection	OL342816	
C18040‐2	2	*P. maydis*	Florida	USA	*Zea mays*	2018	Field collection	OL342817	
C18040‐3	2	*P. maydis*	Florida	USA	*Zea mays*	2018	Field collection	OL342818	
C18069‐1	2	*P. maydis*	Illinois	USA	*Zea mays*	2018	Field collection	OL342819	OL314418
C18069‐2	2	*P. maydis*	Illinois	USA	*Zea mays*	2018	Field collection	OL342820	OL314419
C18069‐3	2	*P. maydis*	Illinois	USA	*Zea mays*	2018	Field collection		OL314420
C19001‐1	2	*P. maydis*	Florida	USA	*Zea mays*	2019	Field collection	OL342821	
C19001‐2	2	*P. maydis*	Florida	USA	*Zea mays*	2019	Field collection	OL342822	
C19001‐3	2	*P. maydis*	Florida	USA	*Zea mays*	2019	Field collection	OL342823	
92794‐1	3	*P. chaetochloae*	Santiago	Dominican Republic	*Setaria* sp.	1931	UIUC Herbarium	OL342860	
92794‐2	3	*P. chaetochloae*	Santiago	Dominican Republic	*Setaria* sp.	1931	UIUC Herbarium	OL342861	
92794‐3	3	*P. chaetochloae*	Santiago	Dominican Republic	*Setaria* sp.	1931	UIUC Herbarium	OL342862	
92812‐1	3	*P. diplocarpa*	California	USA	*Distichilis spicata*	1942	UIUC Herbarium	OL342863	
92821‐1	3	*P. epicampsis*	Arizona	USA	*Muhlenbergia emersleyi*	1948	UIUC Herbarium	OL342864	
92821‐2	3	*P. epicampsis*	Arizona	USA	*Muhlenbergia emersleyi*	1948	UIUC Herbarium	OL342865	
92821‐3	3	*P. epicampsis*	Arizona	USA	*Muhlenbergia emersleyi*	1948	UIUC Herbarium	OL342866	
92825‐1	3	*P. euphorbiaceae*	Mumbai	India	*Euphorbia* sp.	1932	UIUC Herbarium	OL342867	
92825‐2	3	*P. euphorbiaceae*	Mumbai	India	*Euphorbia* sp.	1932	UIUC Herbarium	OL342868	
92825‐3	3	*P. euphorbiaceae*	Mumbai	India	*Euphorbia* sp.	1932	UIUC Herbarium	OL342869	
92845‐3	3	*P. graminis*	Mittelfranken	Germany	*Agropyron repens*	1946	UIUC Herbarium	OL342870	
92922‐3	3	*P. heraclei*	Hessen	Germany	*Heracleum spondylium*	1977	UIUC Herbarium	OL342871	
92925‐2	3	*P. junci*	Holstein	Germany	*Juncus effusus*	1946	UIUC Herbarium	OL342872	
92938‐1	3	*P. maydis*	Arecibo	Puerto Rico	*Zea mays*	1917	UIUC Herbarium	OL342873	
92938‐2	3	*P. maydis*	Arecibo	Puerto Rico	*Zea mays*	1917	UIUC Herbarium	OL342874	
92938‐3	3	*P. maydis*	Arecibo	Puerto Rico	*Zea mays*	1917	UIUC Herbarium	OL342875	
92940‐2	3	*P. maydis*	Arecibo	Puerto Rico	*Zea mays*	1917	UIUC Herbarium	OL342876	
92940‐3	3	*P. maydis*	Arecibo	Puerto Rico	*Zea mays*	1917	UIUC Herbarium	OL342877	
93013‐1	3	*P. rottboelliae*	Luzon	Philippines	*Rottboellia*	1931	UIUC Herbarium	OL342878	
93013‐2	3	*P. rottboelliae*	Luzon	Philippines	*Rottboellia*	1931	UIUC Herbarium	OL342879	
93064‐1	3	*P. sylvatica*	California	USA	*Festuca idahoensis*	1941	UIUC Herbarium	OL342880	
93064‐2	3	*P. sylvatica*	California	USA	*Festuca idahoensis*	1941	UIUC Herbarium	OL342881	
93126‐2	3	*P. vulgata*	Arizona	USA	*Muhlenbergia glauca*	1948	UIUC Herbarium	OL342882	
93126‐3	3	*P. vulgata*	Arizona	USA	*Muhlenbergia glauca*	1948	UIUC Herbarium	OL342883	
BPI638546_1	3	*P. maydis*	Maracas Valley	Trinidad and Tobago	*Zea mays*	1945	USDA Herbarium	OL342884	
BPI638553_1	3	*P. maydis*		Mexico	*Zea mays*	1904	USDA Herbarium	OL342885	
BPI638556_1	3	*P. maydis*	Valle del Cauca	Colombia	*Zea mays*	1929	USDA Herbarium	OL342886	
BPI638558_1	3	*P. maydis*	Antigua	Guatemala	*Zea mays*	1905	USDA Herbarium	OL342887	
BPI638559_1	3	*P. maydis*	Matagalpa	Nicaragua	*Zea mays*	1956	USDA Herbarium	OL342888	
BPI638561_1	3	*P. maydis*	Veracruz	Mexico	*Zea mays*	1932	USDA Herbarium	OL342889	
BPI638564_1	3	*P. maydis*		Mexico	*Zea mays*	1977	USDA Herbarium	OL342890	
BPI638567_1	3	*P. maydis*	Havana	Cuba	*Zea mays*	1918	USDA Herbarium	OL342891	
BPI638568_1	3	*P. maydis*	Alajuela	Costa Rica	*Zea mays*	1947	USDA Herbarium	OL342892	
BPI638570_1	3	*P. maydis*	Vega Baja	Puerto Rico	*Zea mays*	1916	USDA Herbarium	OL342893	
BPI638571_1	3	*P. maydis*	Turrialba	Costa Rica	*Zea mays*	1949	USDA Herbarium	OL342894	
BPI638572_1	3	*P. maydis*	Chimaltenanco	Guatemala	*Zea mays*	1940	USDA Herbarium	OL342895	
BPI638574_1	3	*P. maydis*	Arecibo	Puerto Rico	*Zea mays*	1917	USDA Herbarium	OL342896	
BPI638575_1	3	*P. maydis*	Chimaltenanco	Guatemala	*Zea mays*	1942	USDA Herbarium	OL342897	
BPI638577_1	3	*P. maydis*	Nor Yungas	Bolivia	*Zea mays*	1943	USDA Herbarium	OL342898	
BPI638579_1	3	*P. maydis*		Guatemala	*Zea mays*	1941	USDA Herbarium	OL342899	
BPI638580_1	3	*P. maydis*	Santander	Colombia	*Zea mays*	1936	USDA Herbarium	OL342900	
BPI638581_1	3	*P. maydis*	Vega Baja	Puerto Rico	*Zea mays*	1916	USDA Herbarium	OL342901	
BPI638582_1	3	*P. maydis*	Guatemala	Guatemala	*Zea mays*	1905	USDA Herbarium	OL342902	
BPI638584_1	3	*P. maydis*	Antigua	Guatemala	*Zea mays*	1905	USDA Herbarium	OL342903	
BPI638585_1	3	*P. maydis*		Guatemala	*Zea mays*	1906	USDA Herbarium	OL342904	
BPI638586_1	3	*P. maydis*	Lima	Peru	*Zea mays*	1929	USDA Herbarium	OL342905	
BPI638587_1	3	*P. maydis*	La Vega	Dominican Republic	*Zea mays*	1930	USDA Herbarium	OL342906	
BPI638588_1	3	*P. maydis*		Guatemala	*Zea mays*	1906	USDA Herbarium	OL342907	
BPI893226_1	3	*P. maydis*	Indiana	USA	*Zea mays*	2015	USDA Herbarium	OL342908	
BPI893228_1	3	*P. maydis*	Indiana	USA	*Zea mays*	2015	USDA Herbarium	OL342909	
BPI893230_1	3	*P. maydis*	Indiana	USA	*Zea mays*	2015	USDA Herbarium	OL342910	
BPI893231_1	3	*P. maydis*	Indiana	USA	*Zea mays*	2015	USDA Herbarium	OL342911	
BPI893232_1	3	*P. maydis*	Indiana	USA	*Zea mays*	2015	USDA Herbarium	OL342912	
BPI893233_1	3	*P. maydis*	Illinois	USA	*Zea mays*	2015	USDA Herbarium	OL342913	OL314452
BPI893234_1	3	*P. maydis*	Illinois	USA	*Zea mays*	2015	USDA Herbarium	OL342914	OL314453
BPI910560_1	3	*P. maydis*	Wisconsin	USA	*Zea mays*	2017	USDA Herbarium	OL342915	
BPI910561_1	3	*P. maydis*	Iowa	USA	*Zea mays*	2016	USDA Herbarium	OL342916	
C18046‐1	3	*P. maydis*	Illinois	USA	*Zea mays*	2018	Field collection	OL342829	OL314422
C18046‐2	3	*P. maydis*	Illinois	USA	*Zea mays*	2018	Field collection		OL314421
C18046‐3	3	*P. maydis*	Illinois	USA	*Zea mays*	2018	Field collection		OL314423
C18047‐1	3	*P. maydis*	Illinois	USA	*Zea mays*	2018	Field collection	OL342830	OL314424
C18047‐2	3	*P. maydis*	Illinois	USA	*Zea mays*	2018	Field collection		OL314425
C18047‐3	3	*P. maydis*	Illinois	USA	*Zea mays*	2018	Field collection		OL314426
C18049‐1	3	*P. maydis*	Illinois	USA	*Zea mays*	2018	Field collection		OL314427
C18049‐2	3	*P. maydis*	Illinois	USA	*Zea mays*	2018	Field collection		OL314428
C18050‐2	3	*P. maydis*	Wisconsin	USA	*Zea mays*	2018	Field collection	OL342831	
C18075‐3	3	*P. maydis*	Illinois	USA	*Zea mays*	2018	Field collection	OL342832	OL314429
C18119‐2	3	*P. maydis*	Wisconsin	USA	*Zea mays*	2018	Field collection		OL314430
C18119‐3	3	*P. maydis*	Wisconsin	USA	*Zea mays*	2018	Field collection		OL314431
C18136‐1	3	*P. maydis*	Wisconsin	USA	*Zea mays*	2018	Field collection		OL314432
C18148‐1	3	*P. maydis*	Iowa	USA	*Zea mays*	2018	Field collection	OL342833	OL314433
C18148‐2	3	*P. maydis*	Iowa	USA	*Zea mays*	2018	Field collection	OL342834	OL314434
C18148‐3	3	*P. maydis*	Iowa	USA	*Zea mays*	2018	Field collection	OL342835	OL314435
C18149‐1	3	*P. maydis*	Iowa	USA	*Zea mays*	2018	Field collection	OL342836	OL314436
C18149‐2	3	*P. maydis*	Iowa	USA	*Zea mays*	2018	Field collection	OL342837	OL314437
C18153‐1	3	*P. maydis*	Wisconsin	USA	*Zea mays*	2018	Field collection	OL342838	OL314438
C18153‐2	3	*P. maydis*	Wisconsin	USA	*Zea mays*	2018	Field collection		OL314439
C19007‐1	3	*P. maydis*	Iowa	USA	*Zea mays*	2019	Field collection	OL342839	OL314440
C19007‐2	3	*P. maydis*	Iowa	USA	*Zea mays*	2019	Field collection	OL342840	OL314461
C19007‐3	3	*P. maydis*	Iowa	USA	*Zea mays*	2019	Field collection	OL342841	OL314441
C19008‐1	3	*P. maydis*	Illinois	USA	*Zea mays*	2019	Field collection	OL342842	OL314442
C19008‐2	3	*P. maydis*	Illinois	USA	*Zea mays*	2019	Field collection	OL342843	OL314459
C19008‐3	3	*P. maydis*	Illinois	USA	*Zea mays*	2019	Field collection		OL314443
C19012‐2	3	*P. maydis*	Minnesota	USA	*Zea mays*	2019	Field collection	OL342844	OL314444
C19012‐3	3	*P. maydis*	Minnesota	USA	*Zea mays*	2019	Field collection	OL342845	OL314445
C19022‐2	3	*P. maydis*	Illinois	USA	*Zea mays*	2019	Field collection	OL342846	OL314460
C19022‐3	3	*P. maydis*	Illinois	USA	*Zea mays*	2019	Field collection	OL342847	OL314446
C19025‐1	3	*P. maydis*	Illinois	USA	*Zea mays*	2019	Field collection	OL342848	
C19025‐2	3	*P. maydis*	Illinois	USA	*Zea mays*	2019	Field collection	OL342849	
C19025‐3	3	*P. maydis*	Illinois	USA	*Zea mays*	2019	Field collection	OL342850	
C19040‐1	3	*P. maydis*	Michigan	USA	*Zea mays*	2019	Field collection	OL342851	OL314447
C19040‐2	3	*P. maydis*	Michigan	USA	*Zea mays*	2019	Field collection	OL342852	OL314456
C19040‐3	3	*P. maydis*	Michigan	USA	*Zea mays*	2019	Field collection	OL342853	OL314448
C19043‐2	3	*P. maydis*	Indiana	USA	*Zea mays*	2019	Field collection	OL342854	
C19072‐1	3	*P. maydis*	Wisconsin	USA	*Zea mays*	2019	Field collection	OL342855	OL314449
C19072‐2	3	*P. maydis*	Wisconsin	USA	*Zea mays*	2019	Field collection		OL314450
C19072‐3	3	*P. maydis*	Wisconsin	USA	*Zea mays*	2019	Field collection	OL342856	OL314451
C19106‐1	3	*P. maydis*	Iowa	USA	*Zea mays*	2019	Field collection	OL342857	OL314457
C19106‐2	3	*P. maydis*	Iowa	USA	*Zea mays*	2019	Field collection	OL342858	OL314458
C19106‐3	3	*P. maydis*	Iowa	USA	*Zea mays*	2019	Field collection	OL342859	OL314462
BPI638565	4	*P. maydis*		Venezuela	*Zea mays*	1957	USDA Herbarium	OL342920	
BPI638576	4	*P. maydis*	Mazatenango	Guatemala	*Zea mays*	1906	USDA Herbarium	OL342921	
BPI638583	4	*P. maydis*		Guatemala	*Zea mays*	1907	USDA Herbarium	OL342922	
NC19004‐1	4	*Phyllachora* sp.	New York	USA	*Thinopyrum intermedium*	2019	Field collection	OL342923	OL314463
NC19004‐2	4	*Phyllachora* sp.	New York	USA	*Thinopyrum intermedium*	2019	Field collection		OL314464
NC19004‐3	4	*Phyllachora* sp.	New York	USA	*Thinopyrum intermedium*	2019	Field collection	OL342924	OL314465
NC19026‐1	4	*Phyllachora* sp.	Illinois	USA	*Triticale*	2019	Field collection	OL342917	OL314466
NC19026‐2	4	*Phyllachora* sp.	Illinois	USA	*Triticale*	2019	Field collection	OL342918	OL314467
NC19026‐3	4	*Phyllachora* sp.	Illinois	USA	*Triticale*	2019	Field collection	OL342919	
NC19029‐1	4	*Phyllachora* sp.	Illinois	USA	*Unknown*	2019	Field collection	OL342925	OL314468
NC19029‐2	4	*Phyllachora* sp.	Illinois	USA	*Unknown*	2019	Field collection	OL342926	OL314469
NC19029‐3	4	*Phyllachora* sp.	Illinois	USA	*Unknown*	2019	Field collection		OL314470
NC19030‐1	4	*Phyllachora* sp.	Illinois	USA	*Unknown*	2019	Field collection	OL342927	OL314471
NC19030‐2	4	*Phyllachora* sp.	Illinois	USA	*Unknown*	2019	Field collection	OL342928	OL314472
NC19030‐3	4	*Phyllachora* sp.	Illinois	USA	*Unknown*	2019	Field collection	OL342929	OL314473
NC19032‐1	4	*Phyllachora* sp.	Illinois	USA	*Fall panicum*	2019	Field collection	OL342930	OL314474
NC19032‐2	4	*Phyllachora* sp.	Illinois	USA	*Fall panicum*	2019	Field collection	OL342931	OL314475
NC19032‐3	4	*Phyllachora* sp.	Illinois	USA	*Fall panicum*	2019	Field collection	OL342932	OL314476
NC19034‐1	4	*Phyllachora* sp.	Illinois	USA	*Fescue*	2019	Field collection	OL342933	OL314477
NC19034‐2	4	*Phyllachora* sp.	Illinois	USA	*Fescue*	2019	Field collection	OL342934	OL314478
NC19034‐3	4	*Phyllachora* sp.	Illinois	USA	*Fescue*	2019	Field collection	OL342935	OL314479
NC19035‐1	4	*Phyllachora* sp.	Illinois	USA	*Rye*	2019	Field collection	OL342936	OL314480
NC19035‐2	4	*Phyllachora* sp.	Illinois	USA	*Rye*	2019	Field collection		OL314481
NC19035‐3	4	*Phyllachora* sp.	Illinois	USA	*Rye*	2019	Field collection	OL342937	OL314482
NC19111‐1	4	*Phyllachora* sp.	South Dakota	USA	*Brome grass*	2019	Field collection	OL342938	OL314483
NC19111‐2	4	*Phyllachora* sp.	South Dakota	USA	*Brome grass*	2019	Field collection	OL342939	OL314484
NC19111‐3	4	*Phyllachora* sp.	South Dakota	USA	*Brome grass*	2019	Field collection	OL342940	OL314485
NC19027‐1	5	*Phyllachora* sp.	Illinois	USA	*Triticale*	2019	Field collection	OL342941	OL314486
NC19027‐3	5	*Phyllachora* sp.	Illinois	USA	*Triticale*	2019	Field collection	OL342942	OL314487
NC19028‐1	5	*Phyllachora* sp.	Illinois	USA	*Unknown*	2019	Field collection	OL342943	OL314488
NC19028‐2	5	*Phyllachora* sp.	Illinois	USA	*Unknown*	2019	Field collection	OL342944	OL314489
NC19028‐3	5	*Phyllachora* sp.	Illinois	USA	*Unknown*	2019	Field collection	OL342945	OL314490
NC19033‐2	5	*Phyllachora* sp.	Illinois	USA	*Fall panicum*	2019	Field collection	OL342946	OL314491
NC19037‐1	5	*Phyllachora* sp.	Illinois	USA	*Rye*	2019	Field collection	OL342947	OL314492
NC19037‐2	5	*Phyllachora* sp.	Illinois	USA	*Rye*	2019	Field collection	OL342948	OL314493
NC19037‐3	5	*Phyllachora* sp.	Illinois	USA	*Rye*	2019	Field collection	OL342949	OL314494

^a^ The genetic cluster was determined as a result of the phylogenetic analysis of the combined DNA sequences from the ITS and LSU regions. These are displayed in Figures 2, 3, and Figure S1.

^b^ For contemporary material collected from field samples during this study, specimens of *Phyllachora* from maize were assumed to be *P*. *maydis* and specimens from grass species were treated as unknown *Phyllachora* sp. For herbarium specimens, we included the species name from the herbarium label.

### 2.2 DNA extraction, PCR amplification, and sequencing of stroma from leaf tissue

The DNA of individual stroma not surrounded by a necrotic halo were extracted using the X‐Tract‐N‐AMP kit following manufacturer protocols (Sigma). The complete internal transcribed spacer region of ribosomal DNA (ITS1‐5.8S‐ITS2) with primers ITS1f and ITS4 (White et al., 1990). Stroma without necrotic halos was selected to reduce the potential for contamination by saprophytic fungi that may be present on necrotic tissue within these lesions.

The ITS gene region was amplified from DNA extracted from each stroma using the primer pair ITS1f and ITS4 (Bruns & Gardes, 1993; White et al., 1990) with 35 cycles of the following: 95°C 5 min, 94°C 30s, 52°C 30s, 72°C 1 min, followed by 72°C for 8 min, and a final hold at 4°C in a Thermo Fisher SimpliAmp thermocycler (Thermo Fisher Scientific, Waltham, WA). Individual PCR products from corresponding DNA extractions were loaded into 2% agarose gels and separated via electrophoresis for 40 min at 110V. All gels contained a *P*. *maydis*‐positive control, a *Fusarium graminearum*‐positive control, and a negative buffer control for quality assurance. Bands on gels were visualized using an Axygen gel imaging station (Axygen, Inc., Union City, CA). Stroma of *Phyllachora* spp. can be colonized by or associated with several other fungal species (Hock et al., 1992, 1995; McCoy et al., 2019). Consequently, samples returning a single band between 300 and 500 bp were considered free of additional fungal contaminants and used in subsequent analyses.

DNA from samples returning a single ITS band were subject to amplification of the large ribosomal subunit (LSU) region using the primer pair LR0R and LR5 (Dayarathne et al., 2017) using the aforementioned thermocycler conditions. All PCR products were purified using QIAquick PCR kits (Quiagen, Inc., Hilden, Germany), and the ITS and LSU amplicons for all samples were sequenced in the forward and reverse directions at the University of Illinois Core DNA Sequencing Facility (Urbana, Illinois).

### 2.3 Sequence alignment, phylogenetic analysis, and molecular identification

Sequences generated from this study were combined with sequences obtained from GenBank. *Exserohilum turcicum* and *Cocoicola californica* were selected as the outgroups. Sequence data were aligned and concatenated using MAFFT v.7 (www.mafft.cbrc.jp/alignment/server/) using the G‐INS‐I model and manually inspected. The best = fit partitioning schemes were determined using PartitionFinder (Lanfear et al., 2017) and used to build the phylogenies. Both single gene and concatenated gene sets were analyzed using a maximum likelihood (ML) analysis using RaxML and Bayesian inference with MrBayes. The ML phylogenies were generated by RaxML (Stamatakis, 2014) under GTR model with gamma distributed rate heterogeneity with 1000 bootstrap replicates. For the Bayesian inference, we used MrBayes v. 3.2.6 (Ronquist et al., 2012) using the general time reversible (GTR) model selected for the entire unpartitioned alignment, with the likelihood parameters setting (lset) number of substitution types (nst) = 6, with a proportion of sites invariable and the rest drawn from the gamma distribution (rate = invgamma). Four independent analyses, each starting from a random tree, were run under the same conditions for the combined gene alignment. Three hot and one cold chains of Markov Chain Monte Carlo iterations were performed. Analyses were run with 1,000,000 generations with sampling every 100 generations. The first 250,000 generations were discarded as the chains were converging (burnin period). Resulting trees were visualized with iTOL (Interactive Tree of Life) v.6 (https://itol.embl.de/) or MEGA. Sequences generated in this study were deposited in GenBank (Table 1).

## 3 RESULTS

### 3.1 DNA extraction and PCR amplification from herbarium and contemporary samples

A total of 186 samples from 12 states in the United States (*n* = 130), 4 states in Mexico (*n* = 13), 3 Central American countries (*n* = 13), 4 South American countries (*n* = 6), 4 Caribbean Islands (*n* = 16), Germany (*n* = 3), India (*n* = 3), and the Philippines (*n* = 2) were sequenced and analyzed as noted above. There were varying levels of success for the amplification of each genetic locus among the samples. This was particularly the case for many of the herbarium samples, some of which were more than 100 years old. The ITS region was the most successfully amplified and sequenced, with 168 sequences generated. Whereas 91 sequences were generated for the LSU locus (Table 1).

### 3.2 Phylogenetic diversity of *Phyllachora* isolates infecting maize and grasses

**FIGURE 2:**
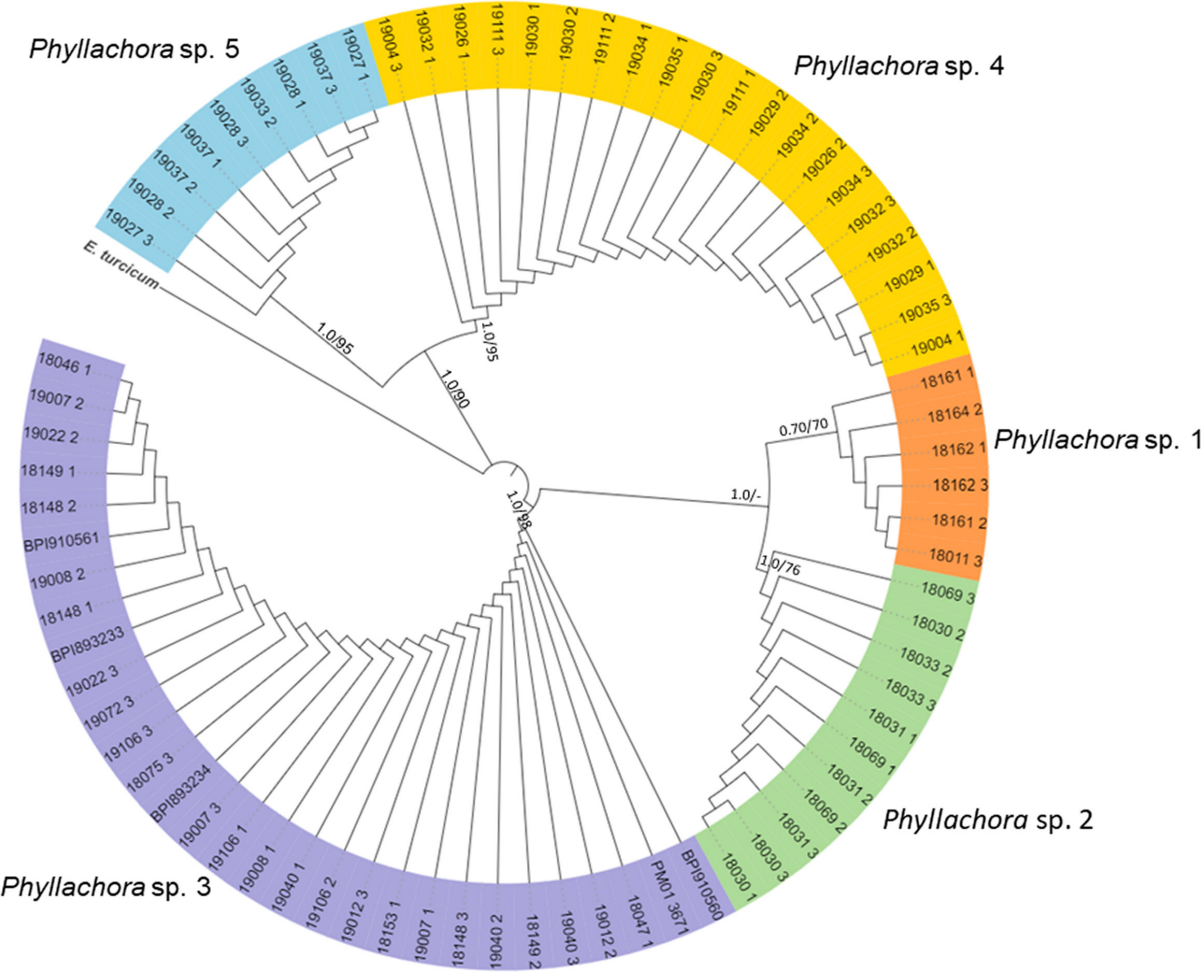
Maximum likelihood phylogenetic tree based on combined ITS and LSU sequence data from the stroma of 76 *Phyllachora* isolates from herbarium and contemporary samples of infected maize and other grass hosts. Values of Bayesian PP > 0.70 or ML BS > 70% are given at nodes at the first and second positions, respectively. *Exserohilum turcicum* CBS 690.71 was used as the outgroup

Based on both ITS + LSU (Figure 2) and ITS (Figure S1) phylogenies, we observed five genetically distinct groups that represent individual species of *Phyllachora* with strong bootstrap and posterior probability support (>70%). The results suggest that tar spot on maize in the United States is caused by three closely related species of *Phyllachora* (Figure 2). In all, four species were found on maize but only *Phyllachora* sp. 1, *Phyllachora* sp. 2, and *Phyllachora* sp. 3 were recovered from contemporary maize in the United States, while *Phyllachora* sp. 4 was recovered from herbarium samples collected in Guatemala and Venezuela (Table 1).

**FIGURE 3:**
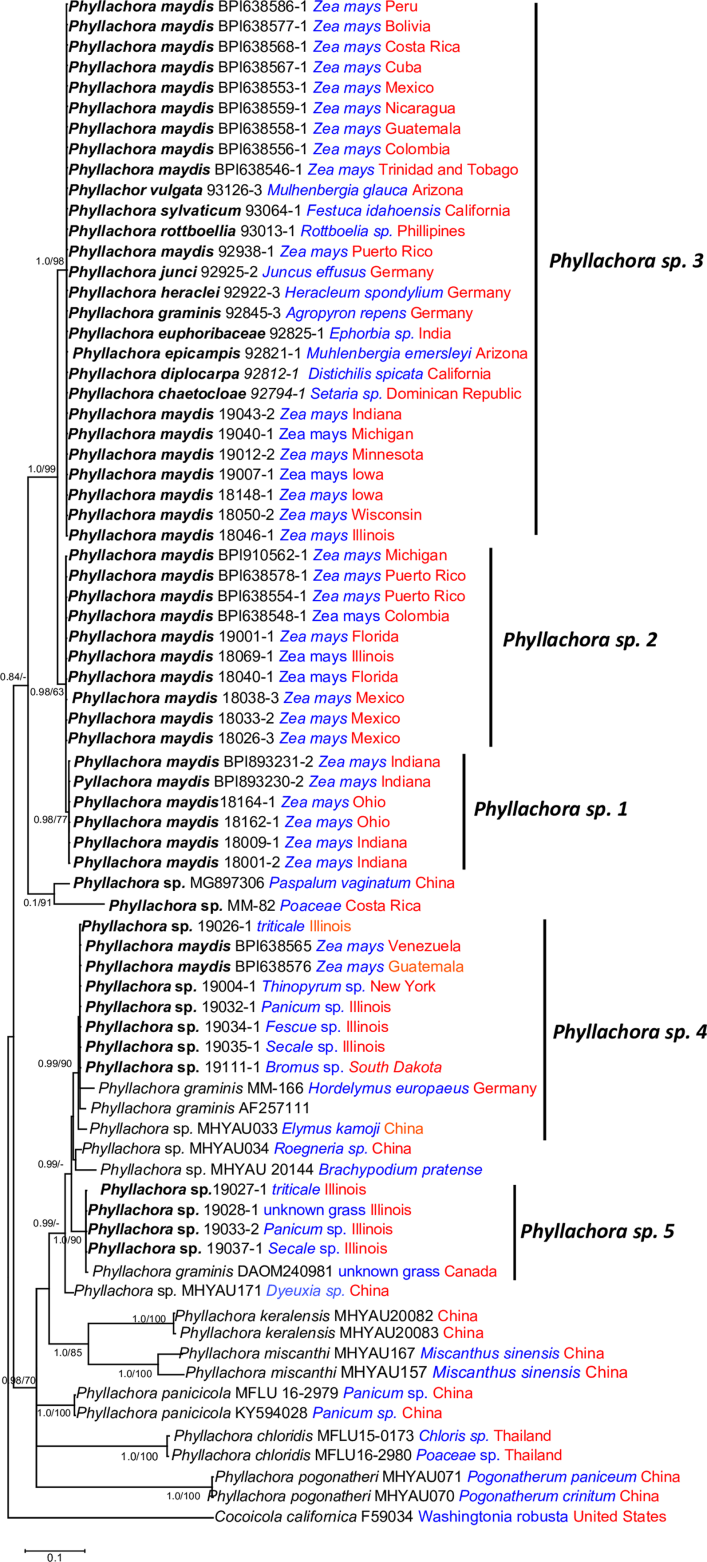
Maximum likelihood phylogenetic tree based on ITS sequence data from geographically representative isolates of the five genetic groups of maize and grass‐infecting *Phyllachora* from this study and *Phyllachora* species available from GenBank. Values of Bayesian PP > 0.70 or ML BS > 70% are given at nodes at the first and second positions, respectively. Isolates sequenced in this project are denoted in bold. Information on host plant is indicated in blue text and geographic origin in red text

Samples of *Phyllachora* sp. 3 represent the broadest geographic and host range and was also the most frequently recovered species associated with *Phyllachora* sp. stroma on maize from both herbarium and contemporary specimens representing a span of time from 1904 to 2019 (Table 1). Samples of *Phyllachora* sp. 3 on maize were reported and recovered from herbarium samples throughout the Americas including Bolivia, Colombia, Costa Rica, Cuba, Dominican Republic, Guatemala, Mexico, Nicaragua, Puerto Rico, and Trinidad and Tobago prior to the first report of tar spot on maize in the United States. Importantly, the isosyntype specimen of *P*. *maydis* (BPI638553) collected in Mexico in 1904 and the *P*. *maydis* isolate (BPI893226) used in the first report of tar spot in the United States in 2015 are both part of *Phyllachora* sp. 3 and isolates of this species have since been recorded in Illinois, Indiana, Iowa, Michigan, Minnesota, and Wisconsin. This represents the widest geographic range of the maize‐infecting *Phyllachora* species in the United States among the samples included in this study. However, isolates of *Phyllachora* sp. 3 were also recovered from another 10 host species including monocots and dicots, with a global distribution including 12 countries across South, Central, and North America and the Caribbean, as well as Germany, India, and the Philippines (Table 1; Figure 3). The herbarium samples associated with each of the 10 host species represented morphologically recognized species of *Phyllachora* including *P*. *graminis*, *P*. *heraclei*, *P*. *junci*, *P*. *chaetochloae*, *P*. *diplocarpa*, *P*. *epicampis*, *P*. *euphorbiaceae*, *P*. *rottboelliae*, *P*. *sylvatica*, *and P*. *vulgata*.

The other two contemporary maize‐infecting species, *Phyllachora* sp. 1 and *Phyllachora* sp. 2, have a more limited observed host and geographic range. Both species were only recovered on maize. *Phyllachora* sp. 1 was only recovered from contemporary maize samples from Indiana and Ohio, whereas *Phyllachora* sp. 2 was found on herbarium specimens from Colombia and Puerto Rico and contemporary specimens from Puerto Rico, Mexico (Guerrero, Oaxaca, Puebla, and Veracruz), and the United States (Florida, Illinois, and Michigan).

The other species recovered from maize was *Phyllachora* sp. 4. However, samples only included herbarium specimens from Guatemala and Venezuela and did not include any contemporary maize specimens. However, *Phyllachora* sp. 4 was commonly found among grasses in the United States that are found in proximity to maize production fields in Illinois, South Dakota, and New York (Table 1). Isolates of *Phyllachora* sp. 4 were recovered from six grass species in four tribes in the United States representing a broad host range across a breadth of genetically diverse grass species. *Phyllachora* sp. 5 was the only species not recovered from maize but was found on many of the same grass species as *Phyllachora* sp. 4, including rye, triticale, and fall panicum (Table 1).

While there was limited *Phyllachora* sequence data in GenBank, we were able to include the ITS sequence of 19 isolates representing six recognized species of *Phyllachora* to determine any relationship between the isolates used in this study and those submitted previously to GenBank (Figure 3). In the case of *Phyllachora* sp. 4, two isolates referred to as *P*. *graminis*, one from *Hordelymus europaeus* in Germany, and one of unknown origin, as well as isolates of *Phyllachora* on *Elymus kamoji* and *Roegneria* sp. from China grouped together with strong Boostrap support (90%) and posterior probability (0.99). There was also an isolate of *P*. *graminis* from an unknown grass in Canada that grouped together with *Phyllachora* sp. 5, and the herbarium specimen of *P*. *graminis* from *Agropyron repens* in Germany from this study grouped in *Phyllachora* sp. 3 (Figure 3). Our results support the findings of previous observations that *P*. *graminis* is a poorly defined polyphyletic species that has often been assigned to tar spot symptom on a variety of grass hosts.

## 4 DISCUSSION

Since *P*. *graminis* was described by Persoon in 1785 as *Sphaeria graminis* and then transferred to the genus *Phyllachora* by Fuckel (1870), over 300 species have been recorded on graminaceous hosts, and many more on non‐grass hosts. However, Parbery (1967) recognized that there are fewer species associated with grasses and established that there were 95 valid graminicolous *Phyllachora* species world‐wide based on morphological characteristics. In the most complete study of *Phyllachora* species in North America, Orton (1944) identified 45 morphological species from more than 100 host species (Orton, 1944). While this likely represents a significant overestimation of the true number of species in North and Central America, it does demonstrate the vast number of hosts on which *Phyllachora* species have been reported. Our results based on both herbarium and contemporary samples of infected hosts indicate that there are far fewer species of *Phyllachora* in the Americas than indicated by Orton (1944) and Parbery (1967), and the species that are present have a greater host range than previously thought. The predominant species in this study, *Phyllachora* sp. 3, has a broad geographic and host range with the capacity to infect maize throughout South, Central, and North America as well as seven grass species and two dicot species. This phylogenetic species also includes isolates of 11 morphologically determined species of *Phyllachora* (*P*. *chaetochloae*, *P*. *diplocarpa*, *P*. *epicampsis*, *P*. *euphorbiaceae*, *P*. *graminis*, *P*. *heraclei*, *P*. *junci*, *P*. *maydis*, *P*. *rottboelliae*, *P*. *sylvatica*, and *P*. *vulgata*) from herbarium samples collected in the Dominican Republic, Germany, India, Mexico, the Philippines, Trinidad and Tobago, and the United States, indicating global distribution of this species. This expanded host range also now complicates the taxonomic status and the name to be retained by this genetic group. An isolate of *P*. *graminis* collected from *Agropyron repens* from Germany was designated as the lectotype specimen for the genus (Clements & Shear, 1931), and the isolate of *P*. *graminis* examined in this study, while not the lectotype was collected from *A*. *repens* in Germany, indicating that *P*. *graminis* may have precedence for the species name of *Phyllachora* sp. 3. This would have ramifications for *P*. *maydis* as well as several other *Phyllachora* species in *Phyllachora* sp. 3 (Table 1; Figure 3) that appear to be synonyms of *P*. *graminis*. This is based on sequence data from the ITS and/or LSU region and further multi‐gene phylogenetic studies of a larger representation of type material from herbaria and contemporary *Phyllachora* samples from additional hosts is needed for a thorough taxonomic assessment of this genus.

The three maize‐infecting species, *Phyllachora* sp. 1, *Phyllachora* sp. 2, and *Phyllachora* sp. 3, have overlapping geographic and host ranges, providing the opportunity for co‐infection and genetic exchange. Co‐infection on the same leaf tissue by *Phyllachora* sp. 3 and *Phyllachora* sp. 1 was observed on four occasions with herbarium samples (BPI893232_1 and BPI893232_2, BPI893231_1 and BPI893231_2, BPI893226_1 and BPI893226_2, BPI893230_1, and BPI893230_2) from three counties in Indiana. A recent fungal community analysis of tar spot lesions on maize found a similar trend with two distinct OTUs occurring on 21 of 22 maize leaf samples from Michigan (McCoy et al., 2019). A similar phenomenon has also been observed in *Albugo candida*, another biotrophic pathogen with a broad host range (McMullan et al., 2015). Races of *A*. *candida* were not able to infect a host on their own but were able to co‐infect with a race‐specific isolate that suppressed host immunity in that host. The offspring of any genetic introgression or recombination resulted in a race with an expanded host range able to infect both plants infected by the parental strains of *A*. *candida*. A whole‐genome comparison of these *A*. *candida* races found a mosaic‐like genome structure with large portions conserved between races, as well as regions with only 89% sequence similarity. This scenario may explain the wide host range and variation in morphology between hosts in *Phyllachora* species. Sexual reproduction in *P*. *maydis* followed by discharge of infective ascospores commonly occurs on corn leaves annually in maize‐producing regions of the United States (Groves et al., 2020; Kleczewski et al., 2019). The presence of multiple maize‐infecting species in the mid‐western United States, and even on a single infected leaf, combined with frequent sexual recombination, ascospore release and infection, could result in novel populations and/or species of *Phyllachora* that are more virulent on maize or that have an expanded host range. This may also explain why *Phyllachora* sp. 3 has such a broad host range, whereas *Phyllachora* sp. 1 and *Phyllachora* sp. 2 were only found on maize. Individual populations may gain the ability to infect a new host but are still able to sexually recombine with the rest of the population on the original host species. Given the geographic overlap of many grass species in Central, South, and North America, small populations of *Phyllachora* sp. 3 may have adapted to infect a novel grass species, while maintaining the ability to recombine with the larger *Phyllachora* sp. 3 complex, resulting in the expansion of the host range without specialization and speciation.

Speciation has likely occurred in instances where geographic isolation of a new host prevented further introgression with the original population. As maize is commonly grown from Argentina to Canada, it represents a common host for which distinct *Phyllachora* populations may infect and recombine resulting in potentially new and more virulent populations that are still part of the same species. It is unclear if geographic or genetic barriers lead to speciation between the closely related *Phyllachora* sp. 1, *Phyllachora* sp. 2, and *Phyllachora* sp. 3, but the significant overlap in host and geography would indicate a genetic barrier. While *Phyllachora* sp. 1 and *Phyllachora* sp. 2 were only recovered from maize, our sampling scheme was strongly biased toward maize. It is possible that *Phyllachora* sp. 1 and *Phyllachora* sp. 2 are present on other grass and non‐grass hosts in Central and North America and were not sampled in this study. These non‐sampled hosts, if only infected by one of the *Phyllachora* species, may represent the isolation that led to adaptation and speciation.

For now the name *Phyllachora maydis* will be retained by *Phyllachora* sp. 3 as the *P*. *maydis* isosyntype material (BPI638553) clustered with this group. However, the presence of three maize‐infecting species, the lack of type material of *P*. *graminis*, and the potential taxonomic synonymy with *P*. *graminis* and several other *Phyllachora* species makes it difficult to determine which of the maize‐infecting species will retain the name *P*. *maydis*. Therefore, we recommend referring to *Phyllachora* sp. 1, *Phyllachora* sp. 2, and *Phyllachora* sp. 3 as the *Phyllachora maydis* species complex until further morphological and multi‐gene phylogenetic studies can properly delineate these species.

In this work, we conducted the most comprehensive assessment of *Phyllachora maydis* reported to date and provided evidence that our understanding of this species and genera is limited and requires significant attention. The reasons for the emergence of tar spot caused by three species of *Phyllachora* that have been present in Central America, Mexico, and the Caribbean for over 75 years are still unclear. Several scenarios may explain the recent emergence and severity of tar spot caused by *Phyllachora* species in the upper Midwest of the United States. While *Phyllachora* sp. 2 and *Phyllachora* sp. 3 have been present in both Mexico and Puerto Rico for the last century, it is possible that when the fungus was able to be dispersed via wind and rain to the United States, it could not overwinter in colder climates and the disease could not become established. In fact, according to the herbarium specimens, *Phyllachora* sp. 3 has been present in the United States since the 1940s in California and Arizona on native grasses but not maize. However, recent studies have demonstrated that *Phyllachora* spp. can overwinter in Illinois (Kleczewski et al., 2019). Shorter and warmer winters due to climate change could be playing a role in the ability of *Phyllachora* species to survive further north in the United States. Changes in climate patterns during the growing season may also have an impact on this disease as increased temperature and precipitation may promote epidemics of this disease. Finally, a change in maize genetics may also play a role in the increased severity of tar spot. Since maize breeding programs were not selecting for resistance to tar spot, any partial resistance that may have been present in U.S. germplasm may have been lost through genetic drift. The loss of this resistance may not have been noticed until *Phyllachora* species arrived in the primary maize‐growing region of the United States. The disease remains of minor importance in Mexico and Central American maize production, as resistance to this disease would be selected for in‐breeding programs. The most likely scenario for the emergence of tar spot in the United States includes a combination of these factors: (1) introduction of multiple species of *Phyllachora* from Mexico, Puerto Rico, or other Central American countries through movement of infected plant tissue or possible long‐distance movement via wind, rain, hurricane/tropical storm system, etc.; (2) change in climate in the Midwestern maize growing region more hospitable to the growth, reproduction, and survival of *Phyllachora* spp.; and (3) lack of resistance in maize germplasm grown in the Midwestern United States.

## CONFLICT OF INTEREST

The authors have declared that no competing interests exist. Mention of trade names or commercial products in this publication is solely for the purpose of providing specific information and does not imply recommendation or endorsement by the U.S. Department of Agriculture. The U.S. Department of Agriculture prohibits discrimination in all its programs and activities on the basis of race, color, national origin, age, and disability, and where applicable, sex, marital status, familial status, parental status, religion, sexual orientation, genetic information, political beliefs, reprisal, or because all or part of an individual's income is derived from any public assistance program. (Not all prohibited bases apply to all programs.) Persons with disabilities who require alternative means for communication of program information (Braille, large print, audiotape, etc.) should contact USDA's TARGET Center at (202) 720‐2600 (voice and TDD). To file a complaint of discrimination, write to USDA, Director, Office of Civil Rights, 1400 Independence Avenue, S.W., Washington, D.C. 20250‐9410, or call (800) 795‐3272 (voice) or (202) 720‐6382 (TDD). USDA is an equal opportunity provider and employer.

## AUTHOR CONTRIBUTIONS

**Kirk Broders:** Conceptualization (equal); Data curation (equal); Formal analysis (equal); Methodology (equal); Project administration (equal); Visualization (equal); Writing – original draft (lead); Writing – review  & editing (equal). **Gloria Iriarte‐Broders:** Data curation (equal); Formal analysis (equal); Methodology (equal); Visualization (equal); Writing – original draft (equal); Writing – review & editing (equal). **Gary C. Bergstrom:** Resources (equal); Writing – review & editing (equal). **Emmanuel Byamukama:** Resources (equal); Writing – review & editing (equal). **Martin Chilvers:** Resources (equal); Writing – review & editing (equal). **Christian Cruz:** Resources (equal); Writing – review & editing (equal). **Felipe Dalla‐Lana:** Resources (equal); Writing – review & editing (equal). **Zachary Duray:** Data curation (equal); Formal analysis (supporting); Investigation (supporting). **Dean Malvick:** Resources (equal); Writing – review & editing (equal). **Darren Mueller:** Resources (equal); Writing – review & editing (equal). **Pierce Paul:** Resources (equal); Writing – review & editing (equal). **Diana Plewa:** Resources (equal); Writing – review & editing (equal). **Richard Raid:** Resources (equal); Writing – review & editing (equal). **Alison E. Robertson:** Resources (equal); Writing – review & editing (equal). **Catalina Salgado‐Salazar:** Formal analysis (supporting); Methodology (supporting); Resources (equal); Writing – original draft (supporting); Writing – review & editing (equal). **Damon Smith:** Resources (equal); Writing – review & editing (equal). **Darcy Telenko:** Resources (equal); Writing – review & editing (equal). **Katherine VanEtten:** Data curation (supporting); Formal analysis (supporting); Investigation (supporting); Resources (equal). **Nathan M. Kleczewski:** Conceptualization (lead); Data curation (supporting); Formal analysis (supporting); Funding acquisition (lead); Methodology (equal); Project administration (equal); Resources (lead); Supervision (equal); Writing – original draft (equal); Writing – review & editing (equal).

## Supporting information

Fig S1

## Data Availability

All DNA sequence data generated by this project were deposited in GenBank as accessions OL314402‐OL314494 and OL342781‐OL342949.

## References

[ece38832-bib-0001] Bruns, T. D. , & Gardes, M. (1993). Molecular tools for the identification of ectomycorrhizal fungi ‐ Taxon‐specific oligonucleotide probes for suilloid fungi. Molecular Ecology, 2, 233–242. 10.1111/j.1365-294X.1993.tb00013.x 7513242

[ece38832-bib-0002] Cannon, P. F. (1991). Revision of Phyllachora and some similar genera on the host family Leguminosae. Mycological Papers, 163, 1–302.

[ece38832-bib-0003] Cannon, P. (1997). Diversity of the Phyllachoraceae with special reference to the tropics. Biodiversity of Tropical Microfungi, 255–278.

[ece38832-bib-0004] Clements, F. , & Shear, C. (1931). The genera of fungi (2nd ed.). Wilson Co.

[ece38832-bib-0005] Dayarathne, M. C. , Maharachchikumbura, S. S. N. , Jones, E. B. G. , Goonasekara, I. D. , Bulgakov, T. S. , Al‐Sadi, A. M. , Hyde, K. D. , Lumyong, S. , & McKenzie, E. H. C. (2017). *Neophyllachora* gen nov (Phyllachorales), three new species of *Phyllachora* from Poaceae and resurrection of Polystigmataceae (Xylariales). Mycosphere, 8, 1598–1625. 10.5943/mycosphere/8/10/2

[ece38832-bib-0006] Francisco, C. S. , Ma, X. , Zwyssig, M. M. , McDonald, B. A. , & Palma‐Guerrero, J. (2019). Morphological changes in response to environmental stresses in the fungal plant pathogen *Zymoseptoria tritici* . Scientific Reports, 9, 1–18. 10.1038/s41598-019-45994-3 31270361 PMC6610121

[ece38832-bib-0007] Fuckel, L. (1870). Symbolae mycologicae. Beiträge zur Kenntniss der Rheinischen Pilze. Jahrbücher des Nassauischen Vereins für Naturkunde.

[ece38832-bib-0008] Groves, C. , Kleczewski, N. , Telenko, D. E. P. , Chilvers, M. I. , & Smith, D. L. (2020). *Phyllachora maydis* ascospore release and germination from overwintered corn residue. Plant Health Progress, 21, 26–30.

[ece38832-bib-0009] Hock, J. , Dittrich, U. , Renfro, B. L. , & Kranz, J. (1992). Sequential development of pathogens in the maize tar spot disease complex. Mycopathologia, 117, 157–161. 10.1007/BF00442777

[ece38832-bib-0010] Hock, J. , Kranz, J. , & Renfro, B. L. (1995). Studies on the epidemiology of the tar spot disease complex of maize in Mexico. Plant Pathology, 44, 490–502. 10.1111/j.1365-3059.1995.tb01671.x

[ece38832-bib-0011] Kleczewski, N. M. , & Bowman, N. D. (2020). An Observation of Corn tar spot dispersal from agricultural fields to an isolated urban plot. Plant Health Progress, 22, 69–71. 10.1094/PHP-10-20-0082-BR

[ece38832-bib-0012] Kleczewski, N. , Donnelly, J. , & Higgins, R. (2019). *Phyllachora maydis*, causal agent of tar sport on corn, can overwinter in Northern Illinois. Plant Health Progress, 20, 178.

[ece38832-bib-0013] Kleczewski, N. M. , Plewa, D. E. , Bissonnette, K. M. , Bowman, N. D. , Byrne, J. M. , LaForest, J. , Dalla‐Lana, F. , Malvick, D. K. , Mueller, D. S. , Chilvers, M. I. , Paul, P. A. , Raid, R. N. , Robertson, A. E. , Ruhl, G. E. , Smith, D. L. , & Telenko, D. E. P. (2020). Documenting the establishment, spread, and severity of *Phyllachora maydis* on corn, in the United States. Journal of Integrated Pest Management, 11, 14.

[ece38832-bib-0014] Lanfear, R. , Frandsen, P. B. , Wright, A. M. , Senfeld, T. , & Calcott, B. (2017). PartitionFinder 2: New methods for selecting partitioned models of volution for molecular and morphological phylogenetic analyses. Molecular Biology and Evolution, 34, 772–773. 28013191 10.1093/molbev/msw260

[ece38832-bib-0015] Maharachchikumbura, S. S. N. , Hyde, K. D. , Jones, E. B. G. , McKenzie, E. H. C. , Bhat, J. D. , Dayarathne, M. C. , Huang, S.‐K. , Norphanphoun, C. , Senanayake, I. C. , Perera, R. H. , Shang, Q.‐J. , Xiao, Y. , D’souza, M. J. , Hongsanan, S. , Jayawardena, R. S. , Daranagama, D. A. , Konta, S. , Goonasekara, I. D. , Zhuang, W.‐Y. , … Wijayawardene, N. N. (2016). Families of Sordariomycetes. Fungal Diversity, 79, 1–317. 10.1007/s13225-016-0369-6

[ece38832-bib-0016] Mardones, M. , Trampe‐Jaschik, T. , Oster, S. , Elliott, M. , Urbina, H. , Schmitt, I. , & Piepenbring, M. (2017). Phylogeny of the order Phyllachorales (Ascomycota, Sordariomycetes): among and within order relationships based on five molecular loci. Persoonia, 39, 74–90. 29503471 10.3767/persoonia.2017.39.04 PMC5832958

[ece38832-bib-0017] McCoy, A. G. , Roth, M. G. , Shay, R. , Noel, Z. A. , Jayawardana, M. A. , Longley, R. W. , Bonito, G. , & Chilvers, M. I. (2019). Identification of fungal communities within the tar spot complex of corn in Michigan via next‐generation sequencing. Phytobiomes Journal, 3, 235–243. 10.1094/PBIOMES-03-19-0017-R 31867561 PMC6923758

[ece38832-bib-0018] McMullan, M. , Gardiner, A. , Bailey, K. , Kemen, E. , Ward, B. J. , Cevik, V. , Robert‐Seilaniantz, A. , Schultz‐Larsen, T. , Balmuth, A. , Holub, E. , van Oosterhout, C. , & Jones, J. D. G. (2015). Evidence for suppression of immunity as a driver for genomic introgressions and host range expansion in races of *Albugo candida*, a generalist parasite. Elife, 4. 10.7554/eLife.04550 PMC4384639 25723966

[ece38832-bib-0019] Money, N. P. (2013). Against the naming of fungi. Fungal Biology, 117, 463–465. 10.1016/j.funbio.2013.05.007 23931113

[ece38832-bib-0020] Morris, C. E. , & Moury, B. (2019). Revisiting the concept of host range of plant pathogens. Annual Review of Phytopathology, 57, 63–90. 10.1146/annurev-phyto-082718-100034 31082307

[ece38832-bib-0021] Mueller, D. S. , Wise, K. A. , Sisson, A. J. , Allen, T. W. , Bergstrom, G. C. , Bissonnette, K. M. , Bradley, C. A. , Byamukama, E. , Chilvers, M. I. , Collins, A. A. , Esker, P. D. , Faske, T. R. , Friskop, A. J. , Hagan, A. K. , Heiniger, R. W. , Hollier, C. A. , Isakeit, T. , Jackson‐Ziems, T. A. , Jardine, D. J. , … Wiebold, W. J. (2020). Corn yield loss estimates due to diseases in the United States and Ontario, Canada, from 2016 to 2019. Plant Health Progress, 21, 238–247. 10.1094/PHP-05-20-0038-RS

[ece38832-bib-0022] Orton, C. R. (1944). Graminicolous species of *Phyllachora* in North America. Mycologia, 36, 18–53. 10.2307/3754878

[ece38832-bib-0023] Parbery, D. G. (1967). Studies on graminicolous species of *Phyllachora* Nke in Fckl. V. A taxonomic mongraph. Australian Journal of Botany, 15, 271–375.

[ece38832-bib-0024] Ronquist, F. , Teslenko, M. , Van der Mark, P. , Ayers, D. L. , Darling, A. , Höhna, S. , Larget, B. , Liu, L. , Suchard, M. A. , & Huelsenbeck, J. P. (2012). MrBayes 3.2: Efficient Bayesian phylogenetic inference and model choice across a large model space. Systematic Biology, 61, 539–542. 22357727 10.1093/sysbio/sys029 PMC3329765

[ece38832-bib-0025] Slepecky, R. A. , & Starmer, W. T. (2009). Phenotypic plasticity in fungi: A review with observations on *Aureobasidium pullulans* . Mycologia, 101, 823–832. 19927747 10.3852/08-197

[ece38832-bib-0026] Stamatakis, A. (2014). RAxML version 8: A tool for phylogenetic analysis and post‐analysis of large phylogenies. Bioinformatics, 30, 1312–1313. 10.1093/bioinformatics/btu033 24451623 PMC3998144

[ece38832-bib-0027] Stockinger, H. , Walker, C. , & Schüßler, A. (2009). ‘*Glomus intraradices* DAOM197198’, a model fungus in arbuscular mycorrhiza research, is not *Glomus intraradices* . New Phytologist, 183, 1176–1187. 10.1111/j.1469-8137.2009.02874.x 19496945

[ece38832-bib-0028] Valle‐Torres, J. , Ross, T. J. , Plewa, D. , Avellaneda, M. C. , Check, J. , Chilvers, M. I. , Cruz, A. P. , Dalla Lana, F. , Groves, C. , Gongora‐Canul, C. , Henriquez‐Dole, L. , Jamann, T. , Kleczewski, N. , Lipps, S. , Malvick, D. , McCoy, A. G. , Mueller, D. S. , Paul, P. A. , Puerto, C. , … Cruz, C. D. (2020). Tar spot: An understudied disease threatening corn production in the Americas. Plant Disease, 104, 2541–2550. 10.1094/PDIS-02-20-0449-FE 32762502

[ece38832-bib-0029] White, T. J. , Bruns, T. , Lee, S. , & Taylor, J. (1990). Amplification and direct sequencing of fungal ribosomal RNA genes for phylogenetics. In M. A. Innis , D. H. Gelfand , J. J. Sninsky , & T. J. White (Eds.), PCR protocols: A guide to methods and applications (pp. 315–322). Academic Press.

