# Supplementary figures and images for: Phyllachora species infecting maize and other grass species in the Americas represents a complex of closely related species
Source: Ecol Evol. 2022 Apr 25;12(4):e8832. doi: 10.1002/ece3.8832 (PMC9036037; doi:10.1002/ece3.8832)

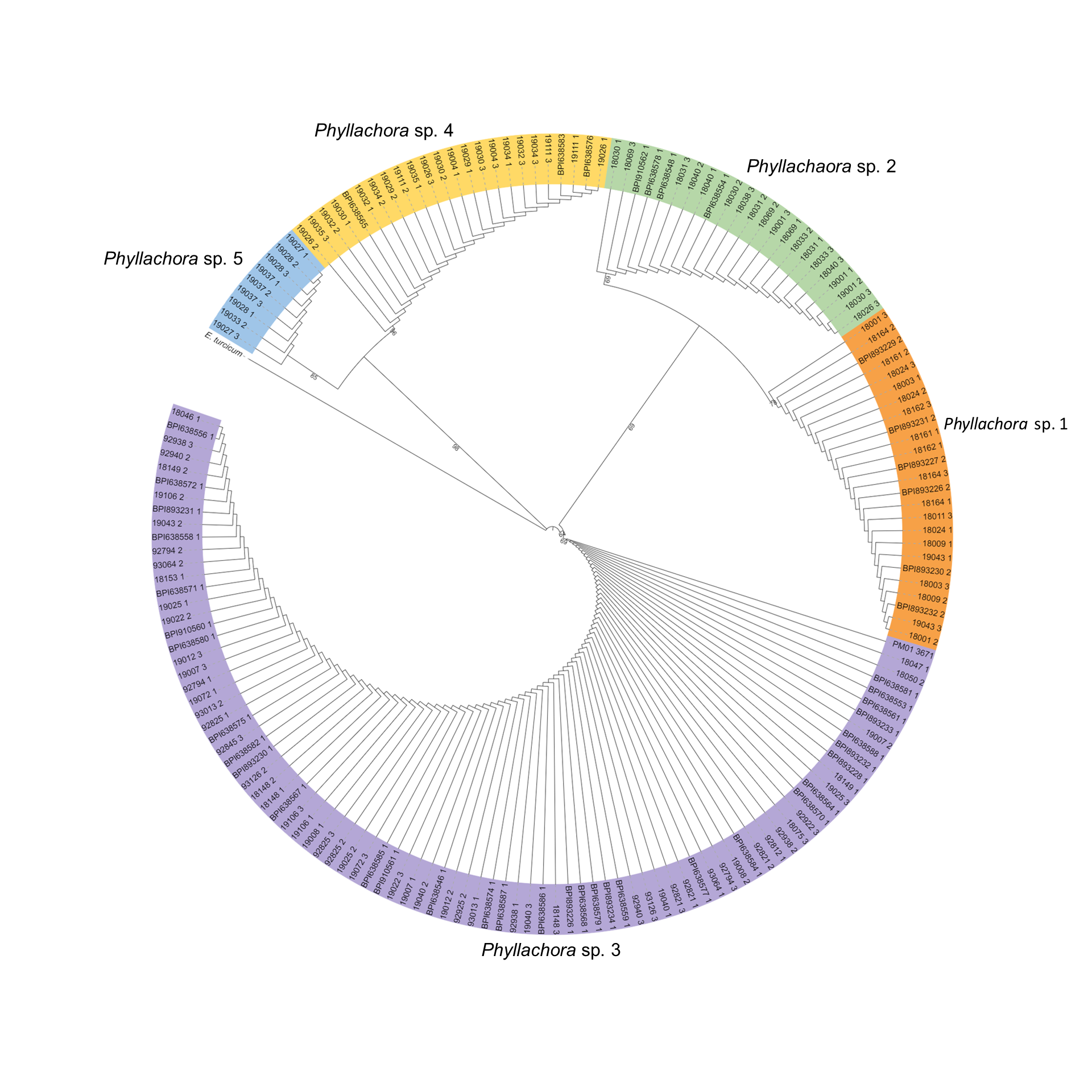

Supplement: Supplementary file 1 — Fig S1 [file ECE3-12-e8832-s001.tif]
